# The Noise Within: Signal-to-Noise Enhancement via Coherent Wave Amplification in the Mammalian Cochlea

**Published:** 2023-07-18

**Authors:** Alessandro Altoè, Christopher A. Shera

**Affiliations:** Auditory Research Center, Caruso Department of Otolaryngology, University of Southern California, Los Angeles, CA 90033

## Abstract

The mammalian inner ear’s extraordinary sensitivity has captivated scientists for decades, largely due to the crucial role played by outer hair cells (OHCs) and their unique piezoelectric properties. These specialized cells, arranged in three rows along the cochlea’s sensory tissue, work in concert to amplify the faintest sounds. Referred to as the “cochlear amplifier,” this mechanism poses a fascinating question: How does it effectively enhance ear sensitivity in real-world scenarios? While simplistic views attribute this enhancement solely to increased cochlear gain, the presence of internal noise in practical settings necessitates a more nuanced approach. Achieving a genuine boost in sensitivity through amplification requires that the signals are amplified more than the internal noise, thus presenting an intriguing challenge. In this study, we analyze the effects of coherent amplification on both signals and internal noise, employing a simple yet powerful mathematical framework and a simplified model of cochlear physics. Our findings not only generalize and expand upon previous discoveries concerning the impact of spatially coherent amplification on signal degradation in active gain media, but also unveil the elegant and efficient wave-based strategy employed by the cochlea to boost ear sensitivity. When considering narrowband signals, this strategy boils down to spatially amplifying the signal within a localized region of the cochlea, followed by rapid attenuation. This location-dependent wave amplification and attenuation meets the necessary conditions for amplifying near-characteristic frequency (CF) signals more prominently than internal noise components of the same frequency. In particular, our analysis reveals that the sharp wave cut-off past the CF location greatly reduces noise contamination, leading us to conclude that the distinctive asymmetric shape of the “cochlear filters” underlies an important but previously unrecognized noise reduction mechanism. When broadening our perspective to encompass broadband signals and noise, the spatially constrained amplification of the different signal components substantially enhances the overall signal-to-noise ratio along the entire length of the cochlea, significantly facilitating detection of broadband signals.

## I. INTRODUCTION

In the 19th century, Bernhard Riemann made a remarkable observation that the sound of a foghorn could be detected from a distance of 5 miles, leading to his conclusion that the human inner ear has the ability to perceive sounds that generate only sub-atomic motions of the eardrum [1]. Over the course of one and a half centuries, Riemann’s conjecture has evolved into an empirical fact [2]. The extraordinary sensitivity of the healthy mammalian ear can be attributed to the piezoelectric behavior of outer hair cells (OHCs) [3], a group of cells arranged in three rows along the sensory tissue (the organ of Corti). Through their coordinated spatial cooperation, these cells magnify the vibrations of the sensory tissue in response to faint sounds by more than two orders of magnitude [4]. The prevailing belief in the field posits that OHCs actively amplify sound-induced waves as they propagate along the spiral structure of the cochlea, a collective mechanism referred to as the “cochlear amplifier” [5]. However, the rationale behind amplification as a viable strategy for enhancing ear sensitivity remains elusive, given that the minimum vibration level required for sensory neurons to detect signals is inherently dictated by the level of internal noise [see e.g. 6]: It remains unclear how the cochlear amplifier, while amplifying signals, can avoid amplifying the accompanying internal noise [7]. Indeed, cochlear internal noise level depends on the same mechanism that control signal amplification [8].

In this study, we investigate the impact of coherent amplification on signals and internal noise through two distinct models: a mathematical model of spatially distributed amplification and an active cochlear model. We begin by examining the simplest scenario, which involves a highly anisotropic (one-dimensional) medium comprising a series of cascaded “noisy” amplifiers where signals propagate in a single direction. We then move to the more complex and biologically relevant case where the medium is nearly isotropic (i.e., when signals propagate in both directions). By analyzing the spatial variation in signal-to-noise ratio (SNR) along these media and their dependence on medium gain, we observe advantageous effects of amplification. Drawing from these findings, we then investigate signal and noise amplification within a simple but physically realistic model of the cochlea. Through this exploration, we gain understanding of how the cochlea’s spatially coherent amplification enhances SNRs, thus boosting the sensitivity of the ear.

## II. SPATIALLY DISTRIBUTED AMPLIFICATION IN NOISY MEDIA

### Propagation of signals and noise in one direction.

**FIG. 1.**
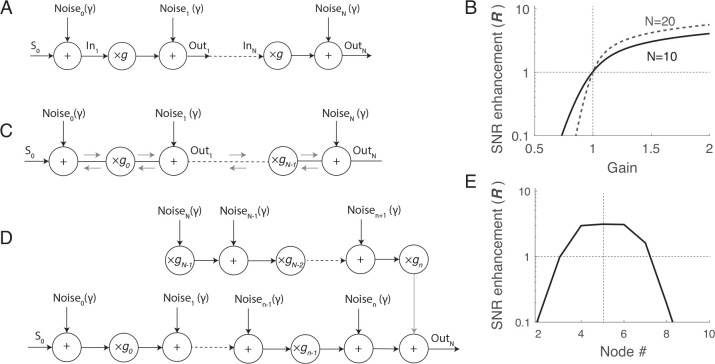
A) Effect of spatially distributed “one-way” amplification on signal and internal noise. The model consists of a chain of linear amplifiers (multipliers) with gain g; the effect of internal noise is simulated by adding noise before and after each amplification stage. B) SNR enhancement (R) at the N-th output of the amplifier chain (shown for N=10 and N=20) as a function of the amplifiers gain g. C) Bidirectional noisy amplification model. In this model, internal noise propagates (while identically amplified) in both directions. D) Equivalent one-way amplification model to study noise and signal response at the n-th node. E) Example of enhancement factor at different nodes in a chain of N=10 bidirectional amplifiers. In this example the amplifiers gain is chosen to improve SNR at node 5 (see text), by imposing $g_{m}=3$ for m<5 and $g_{m}=0.1$ for m≥5.

We start by considering the simpler scenario of the (discrete) distributed “one-way” noisy amplifier, depicted in Fig. 1A. The model consists of a chain of amplifiers that multiply the input signal (S) by a factor g, representing the amplifier gain. The medium’s noise is represented by noise sources that are summed with the propagating signal after each amplification stage. To remove the ambiguity regarding whether noise should be included before or after the amplification stage, the model includes noise sources both at the input and output of the first and last amplifier, respectively. This model approximates a strongly anisotropic medium, where signals and noise propagate only in one direction (from left to right in Fig. 1A). This scenario accurately represents what occurs in many man-made systems, such as cascaded electronic amplifiers or radio repeaters.

In this model we can turn amplification “off” by imposing g=1—and hence model signal propagation in a lossless, noisy medium—or turn it “on” by imposing g>1. When g<1, the distributed amplifiers become effectively distributed “brakes” that attenuate propagating signals. By comparing signal and noise when g=1,g>1 and g<1, we quantify the impact of amplification and attenuation on the SNR along the amplifier’s chain (i.e., at the nodes ${out}_{1,2\ldots n}$ in Fig. 1A.)

The amplitude of the signal at a given node n is simply the amplitude of the input signal passed through n multipliers $\left(\left|S_{n}\right|=g^{n}|S|\right)$ and hence turning on the amplifier boosts the signal amplitude by a gain factor

(1) $$G_{\text{signal}}[n]=g^{n}\text{.}$$

We focus our analysis on the physically relevant case when the noise sources are uncorrelated, meaning that the noise in the medium is spatially incoherent. The main results regarding the differential effects of amplification on signals and internal noise hold even when the noise sources are spatially coherent, and its demonstration is trivial. For simplicity, we assume that the various noise sources are independent versions of the same stochastic process, with a root-mean-square (RMS) amplitude of γ. In this case, the RMS amplitude of the noise $\left(N_{rms}\right)$ at node n can be calculated by incoherent summation (linear summation of power) of the various amplified noise terms. Specifically, the noise power at node n can be expressed as a geometric series, where the m-th term represents the contribution of the (n-m)-th source, amplified (or attenuated) m times. The expression for $N_{\text{rms}}[n]$ can be simplified based on different scenarios:

(2) $$N_{\text{rms}}\left[n\right]=\sqrt{\sum_{m=0}^{n}g^{2m}\gamma }=\{\begin{array}{ll} \sqrt{\frac{g^{2\left(n+1\right)}-1}{g^{2}-1}}\gamma & \;\text{for}\;g\neq 1,\\ \sqrt{n+1}\gamma & \;\text{for}\;g=1. \end{array}$$

Hence, turning on the amplifier boosts the noise gain by a factor of

(3) $$G_{\text{noise}}[n]=\frac{{\left.N_{rms}[n]\right|}_{g\neq 1}}{{\left.N_{rms}[n]\right|}_{g=1}}=\sqrt{\frac{g^{2(n+1)}\;-\;1}{(n\;+\;1)\left(g^{2}\;-\;1\right)}}..$$

The SNR at the node n is given by $R_{n}=\left|S_{n}\right|/\sqrt{2}N_{rms}$, where the factor of $1/\sqrt{2}$ arises from taking the RMS amplitude of the signal. The effect of amplification on the system’s sensitivity can be quantified by the SNR enhancement factor [9]

(4) $$R[n]=R_{n}(\text{on})/R_{n}(\text{off})=G_{\text{signal}}/G_{\text{noise}},$$

where R(on) and R(off) are the SNR with the amplifier on (g≠1) and off (g=1), respectively. Figure 1B illustrates the enhancement factor as a function of g for two values of n. When R>1 the signal is amplified more than the internal noise, resulting in an increase in the SNR at the considered node. Conversely, when R<1, the signal is amplified less than the noise, leading to decrease the SNR at the considered node. It follows from Eqs. (1,3) that amplification (g>1) boosts signals more than internal noise, increasing SNR at all nodes. In particular, the larger the gain, the larger R, resulting in a greater improvement in SNR at any node. Additionally, the longer the chain of amplifiers, the larger the benefit of distributed amplification on the SNR and hence the system’s sensitivity. Conversely, when the amplifiers act as attenuators (g<1), R<1, meaning that the signal is attenuated more than the internal noise. A relevant measure of signal degradation is the noise factor $F_{n}=R_{n}/R_{0}$, which quantifies how the SNR degrades along the transmission line. In our case

(5) $$F_{n}=\sqrt{\frac{g^{2(n+1)}\left(1\;-\;g^{-2}\right)}{g^{2(n+1)}\;-\;1}},$$

which approaches 1 (no significant SNR degradation along the line) when g≫1.

### Signal vs. noise amplification in isotropic media.

To gain a better understanding of the cochlea and similar isotropic media, we now examine the scenario where signals propagate in two directions and the gain can vary along the medium. We simplify the analysis by disregarding potential scattering effects within the medium, and assume that the various noise sources have equal amplitudes (Fig. 1C). In this case, we express the amplification of signals and noise at a given node n by considering the system as a combination of two “one-way” amplification models (Fig. 1D). The propagation of a source from a node n′ to a receiver node n in the model is encapsulated by the discrete Green’s function $G\left[n,n^{\prime }\right]$. In the simplified model, where each node n amplifies the signal by a factor $g_{n}$:

(6) $$G\left[n,n^{\prime }\right]=\prod_{m=\text{min}\left(n,n^{\prime }\right)}^{\text{max}\left(n,n^{\prime }\right)-1}g_{m}+\delta_{n,n^{\prime }},$$

where $\delta_{n,n^{\prime }}$ is the Kronecker delta, and where it can be observed that $G\left[n^{\prime },\;n\right]=G\left[n,n^{\prime }\right]$. In this model, the signal is effectively a source at the node 0 and hence its amplitude at the node n is

(7) $$\left|S_{n}\right|=|S|G[n,\;0].$$

The noise response at node n can be decomposed as the incoherent summation of noise from both the left and right sides of the node:

(8) $$\;N_{\text{rms}}[n]=\sqrt{\sum_{n^{\prime }=0}^{L}{\left(G\left[n,n^{\prime }\right]\gamma \right)}^{2}}=\;=\gamma \sqrt{\sum_{n^{\prime }=0}^{n}{\left(G\left[n,n^{\prime }\right]\right)}^{2}+\sum_{n^{\prime }=n+1}^{N}{\left(G\left[n,n^{\prime }\right]\right)}^{2}}$$

which can be included in the simple “one-way model” by adding the noise contribution from sources located to the right of the considered node (Fig. 1D). In this case, amplification is not necessarily beneficial for the SNR as in the simpler model of Fig. 1A. When the goal is to maximize the SNR at a given node n (for a signal source at node 0), the optimal choice of gain distribution along the transmission line is

(9) $$\begin{array}{r} g_{n^{\prime }}\gg 1\;\text{for}\;n^{\prime }<n\\ g_{n^{\prime }}\ll 1\;\text{for}\;n^{\prime }\geq n. \end{array}$$

**FIG. 2.**
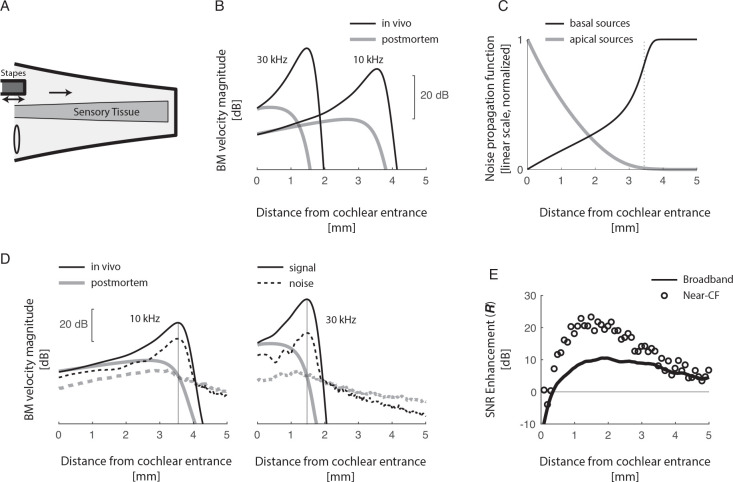
A) Simplified anatomical view of the mammalian cochlea. B) BM magnitude responses in vivo (amplifier on) and post-mortem (amplifier off) to 10 kHz and 30 kHz calculated in a 2D finite difference model of the mouse cochlea. C) Apical and basal noise propagation functions, for narrowband noise centered around of 10 kHz. These functions quantify at each location the expected noise power due to basal and apical noise sources (assuming equal power sources), respectively. D) BM response magnitude to sound signal and narrowband internal noise at 10 and 30 kHz, for a postmortem and in vivo models. The curves are normalized so that the response magnitude to signal and noise is the same postmortem at the CF place—in this way the difference between signal and noise response in vivo visually illustrates that turning on the amplifier boosts SNR at the CF place. E) Enhancement factor (ratio between SNR with amplifier on and amplifier off) along the cochlea, calculated for narrowband near-CF signal and noise, and for broadband signal and noise (white in [4,70] kHz). This figure shows that the near-CF positive SNR enhancement caused by turning on the amplifier, produces a broadband, global SNR enhancement.

In this case, the system approaches the performance of the one-way amplification model at the n-th node. However, differently than in the “one-way” model, it is not possible to boost SNR at all nodes simultaneously (see Fig. 2E).

## III. SIGNAL VS. NOISE AMPLIFICATION IN THE MAMMALIAN COCHLEA

### The cochlear amplifier.

Figure 2A,B illustrates the general function of the mammalian cochlea. Briefly, sound induced vibration of the stapes (the “last” bone of the middle ear) displaces inner-ear fluid, launching hydromechanical waves that propagate slowly from the base (i.e., the entrance) to the apex (the end) of the cochlea. Cochlear wave propagation is frequency dependent, so that waves peak on the BM at different locations depending on frequency—i.e., the cochlea maps frequency into locations, with frequency decreasing from base to apex. The presence of the cochlear amplifier *in vivo* boost waves as they propagate towards their characteristic frequency (CF) place, producing a stronger and more spatially localized response than for passive wave propagation in a dead cochlea. This amplification process effectively narrows the bandwidth of the sensory tissue and the response of auditory neurons, as there is a well-established symmetry between spatial and frequency tuning [10]. By narrowing the bandwidth of the “cochlear filters,” their sensitivity is naturally enhanced through well understood principles [11]. However, the specific benefits of amplification for increasing the ear’s sensitivity remain unclear. It is theoretically possible to narrow the bandwidth of auditory filters through completely passive (non-amplifying) mechanisms [e.g. 12]. Additionally, the nearly isotropic (bidirectional) [13] nature of cochlear amplification further raises questions about its overall impact on global cochlear sensitivity.

Before delving into the effects of amplification in a model, it is worth examining the stereotyped response of the basilar membrane (BM) depicted in Fig. 2B, as it provides an intuitive explanation of how cochlear amplification enhances the ear’s sensitivity. At low sound levels, where the remarkable sensitivity of the ear comes into play, the detection of sound relies on direct activation of the most sensitive auditory neurons [14], which primarily respond to the velocity of the basilar membrane (BM) [15]. In essence, signal detection hinges upon the sensitivity of the sensory tissue that is finely tuned to the frequency of the signal. Thus, given the signal frequency, the challenge of cochlear sensitivity lies in maximizing the SNR at a specific location. The cochlea ingeniously solves this problem—akin to the problem of maximizing SNR at a specific node in the discrete amplifier model discussed earlier—through active traveling wave amplification.

### Amplification of external signals vs. internal sources.

In our analysis of cochlear mechanics, we consider a general model that describes the relationship between the velocity of the sensory organ’s center of mass $\left(V_{CP}\right)$ and the pressure difference across it $\left(P_{0}\right)$ in the linear regime. This relationship is characterized by a phenomenological admittance Y, such that $V=YP_{0}$ (frequency dependencies are not explicitly written for simplicity). Following Newton’s second law and mass conservation we have that [see e.g. 16, and Appendix A]

(10) $$\frac{1}{S}\frac{d}{dx}(S\frac{d\overset{‾}{P}}{dx})+\alpha ZY\overset{‾}{P}=0.$$

In this equation $\overset{‾}{P}$ is the pressure difference between the “upper” and “lower” fluid chamber (see Fig. 2A) averaged over the chambers cross-sectional area (S). The term Z=iωM represents the “longitudinal” impedance accounting for fluid’s effective mass $\left(M\right);\alpha =P_{0}/\overset{‾}{P}$ is a function that relates average and driving pressure [17] depending on wavelength and model’s geometry. For simplicity, we assume one-dimensional (1D) wave propagation, which allows us to set α=1 and $\overset{‾}{P}=P_{0}$. It is worth noting that the equations for two- or three-dimensional (2D and 3D) models are more complex and can be found in Appendix A. However, the qualitative implications derived from the 1D model are well understood and still hold true in more realistic 2D and 3D models, as we will illustrate through numerical simulations [18].

When we assume nearly ideal “reflectionless” boundary conditions at the apical and basal end, we have that the 1D Green’s function is (see Appendix)

(11) $$\begin{array}{l} G\left(x,x^{\prime }\right)\approx \\ \;\;\frac{1}{2i}\sqrt{\frac{S\left(x^{\prime }\right)}{S(x)}\frac{1}{k(x)k\left(x^{\prime }\right)}}\exp\left[-i\int_{\min\left(x,x^{\prime }\right)}^{\max\left(x,x^{\prime }\right)}k(\hat{x})d\hat{x}\right], \end{array}$$

with k the complex wavenumber. The pressure response when the cochlea is driven from the stapes is simply [19]

(12) P(x)=2ik(0)G(x,0).

In the cochlea, the gain per unit length (g) is primarily determined by the imaginary part of the wave number (k) when the spatial gradients of cross-sectional area (S) and k are gentle enough. Specifically, the log-gain per unit length can be approximated as $\frac{d\;log(|G|)}{dx}\sim I(k)$. When I(k)>0, the gain per unit length is larger than one, indicating amplification. On the other hand, when I(k)<0, the gain per unit length is less than one, indicating attenuation. It is important to note that when the cochlear amplifier is inactive, I(k)<0 everywhere. When the amplifier is maximally active, the characteristic frequency (CF) place is approximately located at $\overset{ˆ}{x}$, where I(k)=0, with I(k)>0 when $x<\overset{ˆ}{x}$, and I(k)<0 when $x>\overset{ˆ}{x}$ [20]. Importantly the wave cut-off is dramatic right apically the CF region (see Fig. 2B), so that g≪1 just past $\overset{ˆ}{x}$. In summary, prior to the CF location waves are amplified (g>1), while past the CF location they are rapidly attenuated (g≪1). This arrangement fulfills the conditions for boosting the SNR at the CF place according to the analysis of the bidirectional amplifier [Eq. (9) and Fig. 1C].

### Amplification of narrowband signals and noise.

In the context of analyzing the intrinsic effects of spatial amplification on SNR enhancement, we can focus on a narrow frequency range centered around the characteristic frequency (CF). Within an arbitrarily narrow frequency range, the internal noise can be approximated as spatially incoherent sinusoidal sources with randomly distributed amplitude. The mean of the amplitude distribution is denoted as μ, and the variance as $\sigma^{2}$. By considering this simplified noise model, we can examine the impact of signal amplification on SNR without the confounding effects of bandwidth reduction induced by amplification. The rms noise pressure at a given location x can be approximated as

(13) $${\overset{‾}{P}}_{\text{noise}}(x)\approx \gamma \sqrt{\int_{0}^{L}\;{\left|G\left(x,x^{\prime }\right)\right|}^{2}dx^{\prime }},$$

where $\gamma^{2}=\mu^{2}+\sigma^{2}$. This expression represents the statistical average of the noise pressure considering the amplitude distribution of the incoherent sinusoidal sources. The integral $\int_{0}^{L}\;{\left|G\left(x,x^{\prime }\right)\right|}^{2}dx^{\prime }$ captures the propagation of noise power from basal and apical noise sources to the location x. Assuming that the wavenumber at the cochlear entrance [k(0)] is nearly constant, independent of cochlear amplification, we have that [21]

(14) $$R\propto \frac{\left|G(x,\;0)\right|}{\sqrt{\int_{0}^{x}\;{\left|G\left(x,\;x^{\prime }\right)\right|}^{2}dx^{\prime }\;+\;\int_{x}^{L}\;{\left|G\left(x,{\;x}^{\prime }\right)\right|}^{2}dx^{\prime }}},$$

which represents the ratio between the signal amplification at location x and the square root of the noise power contributions from basal and apical noise sources. In this equation, $\int_{0}^{x}\;{\left|G\left(x,x^{\prime }\right)\right|}^{2}dx^{\prime }$ and $\int_{x}^{L}\;{\left|G\left(x,x^{\prime }\right)\right|}^{2}dx^{\prime }$ represent noise power propagation to the location x from basal and apical noise sources, respectively. The values of these functions, calculated in an active “overturned” 2D model, where the cochlear amplifier is active and the noise is narrowband, centered around 10 kHz are shown in Fig. 2C. The figure shows that at the CF place, the contribution of apical noise sources is negligible compared to that of basal noise sources. This result is consistent with the anticipation that the sharp wave cut-off past the CF place suppresses the contribution of apical noise, making it insignificant. Thus, from the perspective of the CF place, the problem of signal versus noise amplification in the cochlea reduces to the simpler “one-way” amplification model, where amplification boosts the signal more than the internal noise.

Figure 2D, depicts the differential effects of amplification on signal and internal noise in the 2D cochlear model, for frequencies of 30 kHz and 10 kHz. As expected from the analysis of the bidirectional amplifier, turning on the cochlear amplifier boosts the signal more than the internal noise near the CF location. This is evident in the plot, where in vivo the signal amplitude is larger than that of noise near the region where the BM maximally responds [signal and noise levels are normalized so that postmortem they are the same (0 dB) at the CF place]. However, as we move away from the CF location, such as near the cochlear entrance, amplification becomes more pronounced for the internal noise compared to the signal. The differential effect of amplification on signal and internal noise highlights the selective enhancement of the signal relative to the noise at the CF location, where the cochlea achieves optimal sensitivity for sound detection.

### Amplification of broadband signals and noise.

Figure 2E shows the enhancement factor along the cochlea when both signals and noise are broadband—in these simulations signal and noise have white spectrum in the range [4, 70] kHz, covering the range of CF of the cochlear model. Except near the cochlear entrance—where CF waves do not travel long enough to get substantial amplification to the point that there is no SNR enhancement even at CF (open symbols in Fig. 2E)—amplification substantially boosts the broadband SNR, by ~10 dB in the most sensitive locations. These results show that spatially restricted enhancement of the various signals components produces a global increase in cochlear sensitivity to broadband signals.

## IV. DISCUSSION

Despite the inner ear being a biological system with an astounding sensitivity, the fundamental mechanisms underlying it have been largely unexplored. Cochlear mechanical experts typically equate “gain” with “sensitivity,” thus forgetting that the sensitivity of a system depends on internal noise [7]. The handful of attempts in relating cochlear amplification with (true) cochlear sensitivity [e.g. 22, 23] ignore the contribution of wave propagation and rely on non-equilibrium oscillator models whose relevance to cochlear mechanics is uncertain. Here we have shown that established mechanisms of spatially distributed amplification produce significant signal enhancement (Fig. 2E)—not unlike man-made systems such as lasers and active transmission lines [9, 24]. Indeed, by coherently amplifying signals while projecting frequencies into locations, the cochlea employs laser-like narrowband signal amplification [5] to improve sensitivity to both narrow- and broad-band signals [Fig. 2D].

The cochlear waveguide structure is essentially a non-homogeneous transmission line where the cut-off frequency changes with location (or equivalently changes with CF) [25]. In this way, waves within the cochlear frequency range are greatly attenuated before reaching the apical end [see also 26], avoiding noise “build-up” due to scattering from the apical termination which greatly degrade performance of active transmission lines [24]. Our results highlight the functional importance of the asymmetric shape—characterized by a steep high-frequency flank produced by the wave cut-off past the CF place—of the so called “cochlear filters” (i.e., the BM frequency response at one location): near-CF waves coming from more basal locations are amplified, while those coming from more apical locations (where there are noise sources but no signal) are squelched. That is, the cochlear steep wave cut-off underlies a peculiar form of spatial filtering of near-CF components, optimized to reject noise. It is worth noting that the cochlear ear-horn-like geometry contributes significantly to this “optimized spatial filtering”: the cochlear tapered geometry facilitates the propagation of waves from the base to the apex, allowing for efficient signal propagation and amplification [see 19, and Appendix A].

The cochlear signal enhancement strategy elucidated here appears rather simple while biologically robust: cochlear waves are first amplified to then be rapidly attenuated. In this scenario, CF location, spatial amplification and the location where SNR is maximally boosted are intrinsically related— there is no need to assume a priori tight coordination between various frequency and location-dependent mechanisms, but everything follows from the principal mechanism (spatial amplification). Indeed, the fact that the CF location is primarily determined by the location where amplification changes from positive to negative, automatically makes the cochlea operate under the conditions for boosting the SNR at the CF place, regardless of details in the function that describes amplification and its possible perturbations (such as those due to potential “manufacturing errors”).

To conclude, in our model the cochlear amplifier boosts the ear sensitivity to broadband sounds of 6 dB or more, (and significantly more than that for narrowband sounds). As the ear is a pressure sensor, this means that cochlear wave amplification at minimum doubles the distance over which a broadband sound, such as a transient sound caused by a predator’s sudden movement, can be heard—and much more than that in case of narrowband stimuli. Therefore, it is not a great leap of imagination to speculate that the peculiar wave-based frequency analysis performed by the mammalian cochlea might have evolved primarily in response to the selection pressure of extending the range of broadband sound detection.

## Figures and Tables

**FIG. 3.**
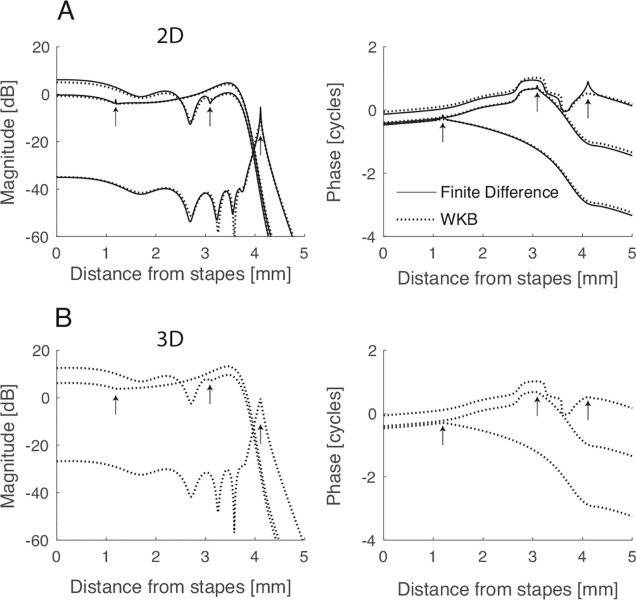
A) Example of Green’s function for a 2D model with reflective basal boundary $\left(\left|R_{\mathrm{st}}\right|\approx 0.14\right)$, calculated numerically in a finite difference model (solid line) or with the WKB approximation [Eqs. (A11,A12), dashed lines]. The source locations for the various curves are indicated with vertical arrows; the source frequency is 10 kHz. B) Approximate Green’s function for a simplified 3D model (see text).

## Appendix A: Green’s function in 1,2 and 3 dimensions

### Equations of motion.

The average pressure difference between the two scalae $\left(\overset{‾}{P}\right)$ and the partition’s center of mass velocity $\left(V_{CP}\right)$ are related by the well-known transmission line equations

(A1)
$$\begin{array}{r} \frac{d\overset{‾}{P}}{dx}=-\frac{i\rho \omega }{S}U,\\ \frac{dU}{dx}=-bV_{CP}, \end{array}$$

with U volume velocity, S the duct’s effective acoustic area, ρ fluid’s density, ω angular frequency and b the partition’s effective width. Partition’s velocity can be expressed as the product of the pressure difference across the tissue $P_{0}$ and a complex admittance $Y_{CP}$

(A2)
$$V_{CP}=P_{0}Y_{CP}=\alpha \overset{‾}{P}Y_{CP}$$

with $\alpha =P0/\overset{‾}{P}$, the short-wave hydrodynamic factor. Combining Eqs. (A1–A2) we find the following expression for $\overset{‾}{P}$

(A3)
$$\frac{1}{S}\frac{d}{dx}(S\frac{d\overset{‾}{P}}{dx})+k_{x}^{2}\overset{‾}{P}=0,$$

with $k^{2}=\alpha ZY$ and $Z=\frac{i\rho \omega }{S}$ the acoustic impedance of the scalae.

### 1D models.

In 1D models, the pressure field is a function of longitudinal distance from the stapes (x) so that $\overset{‾}{P}\equiv P_{0}$ and α=1. The Green’s function $G_{1D}\left(x,x^{\prime }\right)$ is the response to a unitary point pressure source at $x^{\prime }$, and hence can be expressed as

(A4)
$$\frac{1}{S}\frac{d}{dx}(S\frac{dG_{1D}}{dx})+k_{x}^{2}G_{1D}=-\delta \left(x-x^{\prime }\right),$$

with k wavenumber and δ the dirac delta function, and where the somewhat cumbersome notation of defining $G_{1D}$. Note that the pressure source has unit of pressure over length square. We assume reflections boundary conditions and calculate $G_{1D}$ using the Wentzel-Kramers-Brillouin (WKB) approximation. To do so we make a change of variable in Eq. (A4). In particular, we define

(A5)
$$\chi =S(0)\int_{0}^{x}\frac{dx}{S(x)},$$

and $\overset{ˆ}{k}=\frac{S}{S0}k_{x}$. Eq. (A4) can be then rewritten as

(A6)
$$\begin{array}{l} \frac{d^{2}G_{1D}}{d\chi^{2}}+k^{2}G_{1D}=\;-\frac{S^{2}}{S^{2}(0)}\delta \left(x-x^{\prime }\right)=\\ \;=-\frac{S\left(x^{\prime }\right)}{S(0)}\delta \left(\chi -\chi^{\prime }\right), \end{array}$$

In the χ domain the 1D green’s function is [25]

(A7)
$$G\left(\chi ,\chi^{\prime }\right)=\frac{1}{2i}\sqrt{\frac{1}{\hat{k}\left(\chi \right)\hat{k}\left(\chi^{\prime }\right)}}\text{exp}\left[-i\int_{\text{min}\left(\chi ,\chi^{\prime }\right)}^{\text{max}\left(\chi ,\chi^{\prime }\right)}\hat{k}\left(\hat{\chi }\right)d\hat{\chi }\right].$$

Accounting for the source amplitude in the χ domain [Eq. (A6)], and converting the solution back in the x domain we obtain

(A8)
$$\begin{array}{l} G_{\text{1D}}\left(x,\;x^{\prime }\right)=\\ \;=\frac{1}{2i}\sqrt{\frac{S\left(x^{\prime }\right)}{S\left(x\right)}\frac{1}{k\left(x\right)k\left(x^{\prime }\right)}}\text{exp}\left[-i\int_{\text{min}\left(x,x^{\prime }\right)}^{\text{max}\left(x,x^{\prime }\right)}k\left(\hat{x}\right)d\hat{x}\right]. \end{array}$$

### 2D and simplified 3D models.

A tapered 2D “box” model can be physically interpreted as a model where the cross-sectional area of the duct is a rectangle with constant width and varying height, while the partition spans the entire cochlear width and moves up and down as a piston (“wall-to-wall carpeting”, see [25]). The equations for a 2D model, are approximately valid for a 3D model where the cross-sectional shapes of cochlear duct and cochlear partition are circular. When the radius of the partition is sufficiently small pressure is approximately a function of distance from the stapes and x and distance from the partition’s centers (r)—i.e., under these assumptions the 3D model is effectively 2D in cylindrical coordinates [27, 28].

Importantly, although the 2D box model equations are valid for a 3D cylindrical model, the parameters and their spatial gradients are different between the two models [see also 29]. While the partition’s admittance can be strategically chosen so that k is the same in 2 and 3D, [30] the spatial gradient of the cross sectional area S (which determines an important geometric pressure gain factor [19]) differs in the two models—in the tapered box model S∝H, while in the 3D cylindrical model $S\propto H^{2}$, with H scala height (or radius).

Keeping in mind these important caveats, we now proceed to heuristically determine the reduced 2D Green’s function ${\overset{‾}{G}}_{2D}\left(x,x^{\prime }\right)$ which described the average pressure at x in response to a 2D source placed at the center of the partition, i.e., at y=0. Following [25], we note that the 2D reduced Green’s function must obey the following relation

(A9)
$$\frac{1}{S}\frac{d}{dx}(S\frac{d{\overset{‾}{G}}_{2D}\left(x,x^{\prime }\right)}{dx})+k_{x}^{2}{\overset{‾}{G}}_{2D}\left(x,x^{\prime }\right)=ℱ\delta \left(x-x^{\prime }\right),$$

where ℱ, a function to be determined, accounts for the fact that the source is 2D (differently than in the 1D model). Following the results of [25] obtained in a box model of constant cross-sectional area, we have that ${\left.ℱ\right|}_{x^{\prime }}\propto \alpha \left(x^{\prime }\right)$. Because in our tapered model the area changes with location, we further need to figure out if there are systematic differences between 1, 2 and 3D sources that change with the cross-sectional area. In this regard, we note that a 2D point source is $s_{2D}=\delta \left(x-x^{\prime }\right)\delta (y)$ while a one-dimensional one is $s_{1D}=\delta \left(x-x^{\prime }\right)$; their source strength, averaged over the cross sectional area of a two-dimensional model is a factor $H\left(x^{\prime }\right)$ larger in 1 than in 2D [31]. Likewise a 3D source is $s_{3D}=\delta \left(x-x^{\prime }\right)\delta (y)\delta (z)$ whose strength is $S\left(x^{\prime }\right)$ smaller than a 1D one.

Based on these consideration, and further noting that $S\left(x^{\prime }\right)\propto H\left(x^{\prime }\right)$ in 2D (so that we can write equations that are valid in 2D and 3D models), we conclude that $ℱ\approx \alpha \left(x^{\prime }\right)/S\left(x^{\prime }\right)$:

(A10)
$${\overset{‾}{G}}_{2D}\left(x,x^{\prime }\right)\approx \frac{\alpha \left(x^{\prime }\right)}{S\left(x^{\prime }\right)}G_{1D}\left(x,x^{\prime }\right).$$

We now define ${\overset{ˆ}{G}}_{2D}\left(x,x^{\prime }\right)=G_{2D}\left(x,\;0,\;x^{\prime },\;0\right)$ where $G_{2D}\left(x,\;y,x^{\prime },y_{0}\right)$ is the “true” 2D Green’s function, i.e., the function that describes the pressure response at x, y to a unit point source at $x^{\prime }$, $y^{\prime }$. Exploiting the definition of α we have that

(A11)
$$\begin{array}{l} {\hat{G}}_{2\text{D}}\left(x,x^{\prime }\right)\approx \\ \;\frac{\alpha \left(x\right)\alpha \left(x^{\prime }\right)}{2i}\sqrt{\frac{1}{k\left(x\right)k\left(x^{\prime }\right)S\left(x^{\prime }\right)S\left(x\right)}}\text{exp}\left[-i\int_{\text{min}\left(x,x^{\prime }\right)}^{\text{max}\left(x,x^{\prime }\right)}k\left(\hat{x}\right)d\hat{x}\right], \end{array}$$

where it can be appreciated that ${\overset{ˆ}{G}}_{2D}\left(x,x^{\prime }\right)={\overset{ˆ}{G}}_{2D}\left(x^{\prime },\;x\right)$. The same Green’s function holds for the simple 3D model $\left[G_{3D}\left(x,x^{\prime }\right)\approx {\overset{ˆ}{G}}_{2D}\left(x,x^{\prime }\right)\right]$, keeping in mind the caveats regarding the cross-sectional areas in the two models.

### Non-ideal boundary conditions and numerical solutions.

The solution for the Green’s function shown above, are obtained under the assumptions that there is no significant scattering from the basal and apical boundary. While this is a good approximation for the apical boundary—travelings waves are dramatically attenuated before reaching it [26]— the same is not true for the basal boundary, where any impedance mismatch at the stapes has the effect of backscattering a significant fraction of wave power [32]. When the wave frequency is sufficiently lower than the CF near the stapes (so we can assume long-wave behavior near the stapes), we can easily add back the effect of wave scattering and calculate the WKB approximation for this non-idealized Green’s function

(A12)
$$\begin{array}{l} \;{\tilde{G}}_{2\text{D}}\left(x,x^{\prime }\right)={\hat{G}}_{2\text{D}}\left(x,x^{\prime }\right)+\\ +R_{\text{st}}{\bar{G}}_{2\text{D}}\left(0,x^{\prime }\right)\alpha (x)\sqrt{\frac{S(0)k(0)}{S(x)k(x)}}\exp-i\int_{0}^{x}k(\hat{x})d\hat{x}, \end{array}$$

where $R_{\text{st}}$ is the complex reflectance of the stapes [32]. The second term on the right side of Eq. (A12) represents a traveling from the base to the apex, generated by the pressure reflected from the stapes $\left[R_{st}{\overset{‾}{G}}_{2D}\left(0,x^{\prime }\right)\right]$.

### Numerical and semi-analytical calculations

We cross-checked the quality of our calculations by comparing 2D WKB approximation of the Green’s function against numerical calculations performed in a 2D finite-difference tapered model [33, 34], some of which are shown in Fig. 3A. Because calculating the WKB approximation of α requires iterative methods that introduce various inaccuracies, we calculate α numerically, driving the finite-difference model from the stapes. Figure 3B shows the WKB solution of the Green’s function for a 3D model with same wavenumber (k) and height (H) of the 2D model in Fig. 3A. While the agreement between WKB approximation and numerical solution is in most cases excellent, the WKB approximation can introduce spurious but egregious errors (due to the non-uniqueness of the WKB solution in the cut-off region [35]), making calculations noisy, especially at high frequencies. For this reason, in the main text we present results obtained with the 2D finite difference model— the difference between 2D and 3D models are relatively minor, although it is worth mentioning that in 3D the enhancement factors are slightly larger thanks to the more dramatic tapering of the cross-sectional area in 3D than in 2D models.

## Appendix B: Modeling details

We performed all calculations using an overturned model of the mouse cochlea [28], whose parameters are the same used in [13]. Differently than in classic models where the organ of Corti are doesn’t deform, in this model the transverse (up-down) velocity of the center of mass is $V_{CP}=\left(V_{BM}+V_{\text{top}}\right)/2$, where $V_{BM}$ and $V_{\text{top}}$ indicate the velocity of the bottom (BM) and the topside (the reticular lamina and tectorial membrane) of the organ of Corti—their differential velocity is $V_{\text{int}}=V_{\text{top}}-V_{BM}$. Postmortem $V_{BM}$ and $V_{\text{top}}$ are similar so that to a first approximation $V_{\text{int}}\approx 0$ in a passive cochlea, while $V_{\text{int}}\neq 0$ in vivo. The organ of Corti velocity can be rewritten in the compact form $V_{CP}=V_{BM}+V_{int}/2$, where $V_{\text{int}}$ is attributed to the piezo-electric action of OHCs, and is effectively the (velocity) source of wave amplification in the model.

Because the BM stiffness is about one order of magnitude larger than that of the structures surrounding the OHCs, OHC forces produce large displacements of the top side of the organ of Corti while having secondary effects on local BM motion [36, 37]. We therefore assume that internal OHC forces have negligible effects on BM motion so that the mechanical admittance of the BM $\left(Y_{BM}=V_{BM}/P_{0}\right)$ is constant, independent of whether the cochlear amplifier is turned “on” or “off”. For simplicity we assume that $Y_{BM}$ represents the admittance of a damped harmonic oscillator, and exploiting the relation between $V_{CP}$, $V_{BM}$ and $V_{int}$ we express the admittance of the organ of Corti admittance as $Y_{CP}=Y_{BM}\left(1+0.5V_{int}/V_{BM}\right)$. Following previous results we assume that in vivo at low sound levels $0.5V_{\text{int}}/V_{BM}\approx i\beta \tau$ with β=f/CF normalized frequency and τ a (real) constant. Following [19] we assume that $Y_{CP}$ is scaling symmetric (identical when expressed as a function of normalized frequency β) everywhere in the cochlea [38].
